# Role of femoral anterior Bow in cephalomedullary nailing: finite element analysis and New index estimation

**DOI:** 10.1186/s12893-016-0183-9

**Published:** 2016-09-26

**Authors:** Dong Ren, Yueju Liu, Ming Li, Zhaohui Song, Shuangquan Yao, Pengcheng Wang

**Affiliations:** Orthopaedic Traume Service Center, Third Affiliated Hospital to Hebei Medical University, Shijiazhuang, 050051 Hebei Province China

**Keywords:** Femoral anterior bow, The tangent value of the femoral anterior bow, Finite element, Femoral fracture, Femoral cephalomedullary nail

## Abstract

**Background:**

Cephalomedullary Nail (CMN) is seen as the mainstay of internal fixation in femoral fractures. The purpose of the study was to present an accurate calculation method by simulating diverse anterior bow femoral models with finite element software. We hypothesized that anterior cortical penetration in distal femur would occur in patients whose femoral anterior bow was identified as too large for nailing by preoperative measurement of contralateral X-ray.

**Methods:**

A 31-year healthy male was selected for building 3D bone model through computed tomography (CT) scan of right femoral femur. In Creo Parametric 2.0, the middle section of the femur was twisted gradually to simulate the different femoral anterior bow. Ratio of chord height and half chord length, belonging to the middle section arc, was defined as the tangent value of the femoral anterior bow. The value corresponding to the penetration of the CMN at the distal femur was regarded as critical value, showing the extreme curvature for CMN.

**Result:**

Three types of right femoral CMNs (ø10, 11, 12 mm × 350 mm; Smith-Nephew Co.) were involved in our study. The CMN passed through distal femur anterior cortex when the tangent value of the femoral anterior bow are 0.140185, 0.133073, 0.092415 respectively, and the corresponding central angle are 21.72°, 20.92°, 16.32°.

**Conclusions:**

The tangent value of the femoral anterior bow would be a precise calculated method, that eliminate the deviation by description of ratio rather than length of radius. An application of this preoperative evaluation can aid surgeons during surgical planning.

**Trial registration:**

Retrospectively registered.

## Background

Cephalomedullary nailing (CMN) is a widely used technique in the management of femoral fractures; [1] including fractures located beneath the less trochanter, 9 cm above the knee joint, particularly when the fragments are multi-section or smashed. Moreover, the technique was found to be associated with beneficial curative effects in nonunion [2].

The anterior bow of the femur can affect the insertion of CMN [3]. Ostrum et al. [4] described anterior cortical perforation at the distal femur in three patients during nailing of subtrochanteric femur fracture. In these patients, the intramedullary canals with osteoporotic bone were less likely to accommodate relatively straight hip nails. Thus, in order to reduce the complications of nailing, it was proposed that the curvature of intramedullary nails should be increased to accommodate relatively bent medullary. We also encountered challenges related to the insertion of intramedullary nail during operation.

The purpose of this study was to investigate the influence of femur anterior bow on nail insertion by simulating different radius of the femur anterior bow with the finite element method and to device the concept of the tangent value of femoral anterior bow as a convenient evaluation method to express the curvature of the femur. Furthermore, we formulated treatment strategies in accordance with the measurement and calculation of the preoperative X-ray of the contralateral femur. That means plate devices or short nails were preferred rather than long CMNs when the penetration was predicted.

## Methods

A right femur from a 31-year-old healthy male volunteer was used for the study. The study was approved by the ethics committee prior to the application of radioactive techniques. A complete 3D model of cortical and cancellous bone was prepared using the right femoral CT scan. Three types of right femoral reconstruction nails were used (ø10, 11, 12 mm × 350 mm; Smith-Nephew Co.), and the proximal femoral screw (ø6.4 mm × 85 mm) was prepared using rough function of parameterized modeling software Creo Parametric 2.0 (Parametric Technology Corporation, PTC, USA).

**Fig. 1:**
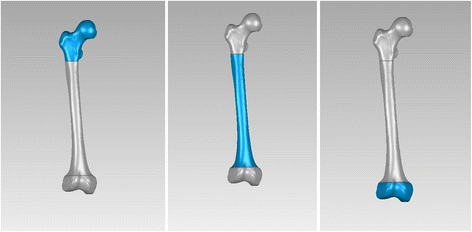
Sections of the femoral model sliced by two planes generated in Geomagic Studio 12.0

**Fig. 2:**
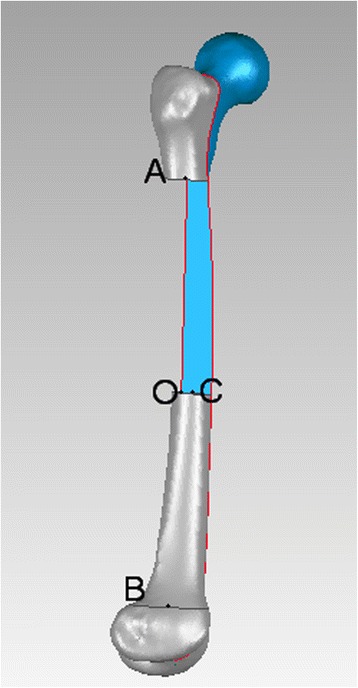
Lateral view of femoral and segment marks

**Fig. 3:**
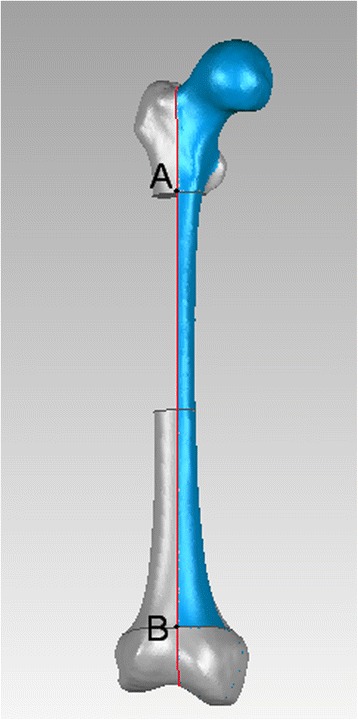
AP view of femoral and segment marks

**Fig. 4:**
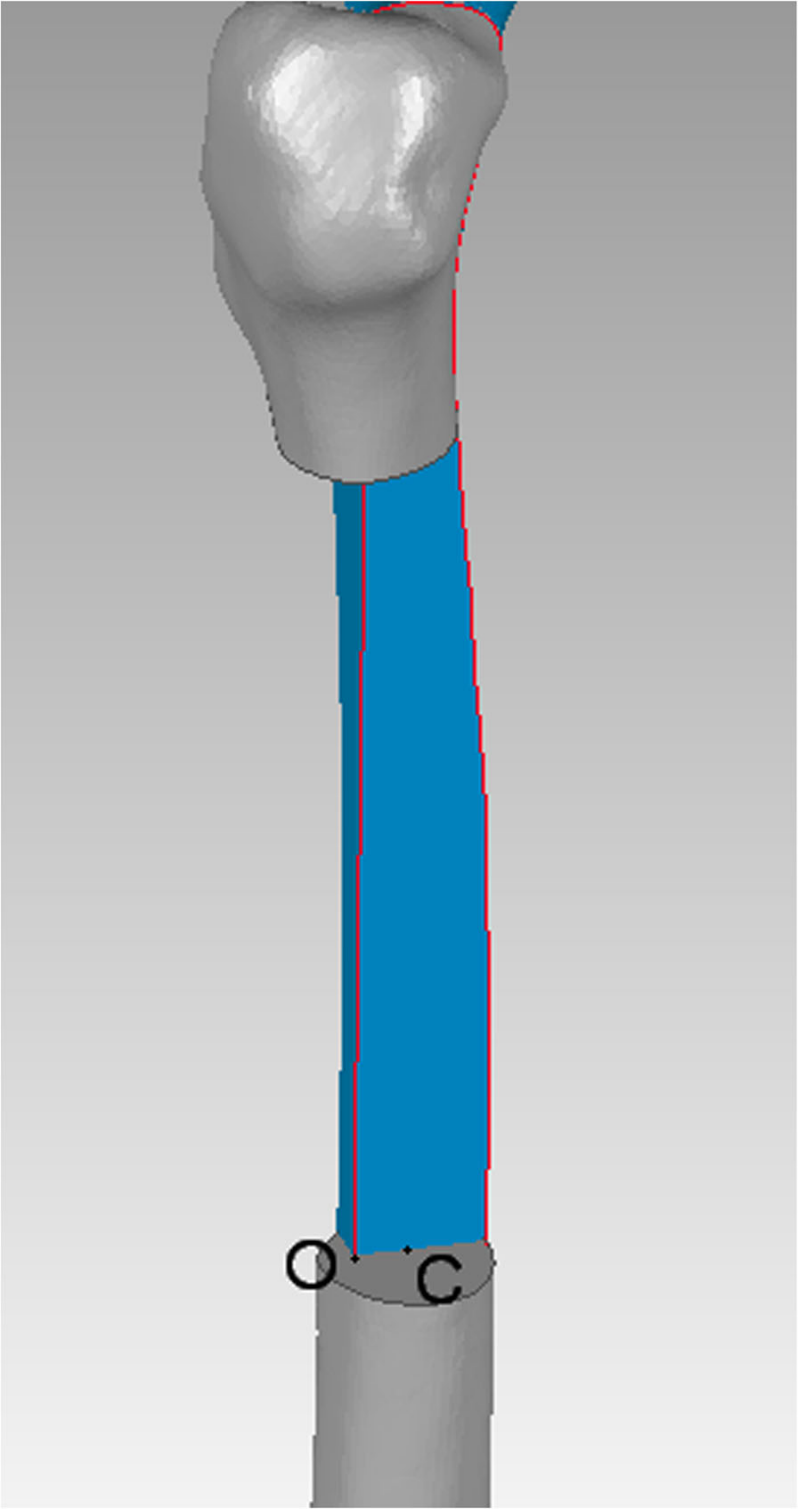
Feature of middle section

As it is defined, both posterior edges of medial and lateral femoral condyles overlapping in the lateral view, and the femur rotated 90° with the bone length axis based on lateral view we get anteroposterior (AP) view. A 3-point circle function in the program, which utilizes these points to calculate the radius of the circle, was used to determine the femoral radius of the curvature. These points were the midpoints of three planes across the femur: first plane immediately beneath the lesser trochanter in the AP view, second plane immediately above the flare of the condyles in the lateral view (just across the adductor tubercle), and third plane midway between the first and second lines. The femur was sliced into three sections by the first and second planes (Fig. 1). Two mutually-perpendicular planes (equivalent to slightly tilted coronal and sagittal) were established that intersect on the line AB. The points: A, B were located in the middle of first and second planes in the AP and lateral view, respectively. (Fig. 2, 3) Established a new plane (equivalent to slightly tilted horizontal plane) perpendicular to the line AB just across point O (Fig. 4),the middle point of line AB. Point C located in the line which the the horizontal and sagittal plane intersect on, as the middle point of middle section femoral cortex in the lateral view. Following the measurement, the initial femoral middle section radius of curvature and the central angle in the lateral view was obtained.

**Fig. 5:**
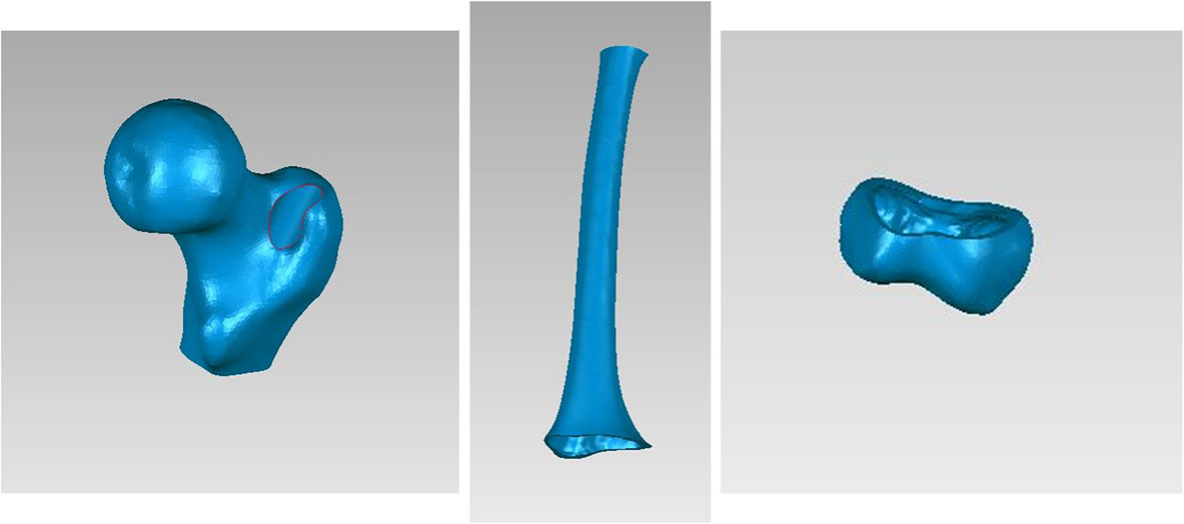
Establishment of the insertion channel of intramedullary nail

Three solid digital stereolithography structures were created to represent: proximal tunnel, consisting of the top of medullary and a 14 mm passageway from the entrance toward the medullary; medium tunnel, which was established on the basis of the inherent medullary; and distal tunnel, filling the distal femoral section except the cortical bone. Three passageways with three diameters (11/12/13 mm) were manufactured along the anterior bow to simulate the conditions after intramedullary reaming near the femoral isthmus. Distal cancellous bone was disposed as medullary (Fig. 5). Tunnels of these three sections integrated the insertion path, which accommodated the 350 mm cephalomedullary nail.

The proximal 14 mm tunnel was created by reaming to adopt the 13 mm nail tail. Of the three establishments, the middle, 12 mm tunnel, was obtained based on the initial medullary nailing. The initial medullary cavity was transferred to Geomagic Studio12.0 software for offsetting −0.5 mm processing in order to reduce the medullary cavity by 1 mm to establish the 11 mm tunnel. Similarly, the establishment of the 13 mm channel was carried out by offsetting +0.5 mm process.

In Creo Parametric 2.0, the middle section of the femur was twisted so as to obtain the femur models with different radius of the femoral anterior bow. The process of nail insertion into the channel in the femur model was simulated, the relationship between the nail and femoral medullary cavity wall was observed by redefining the femoral anterior bow and spacing position of the nail.

**Fig. 6:**
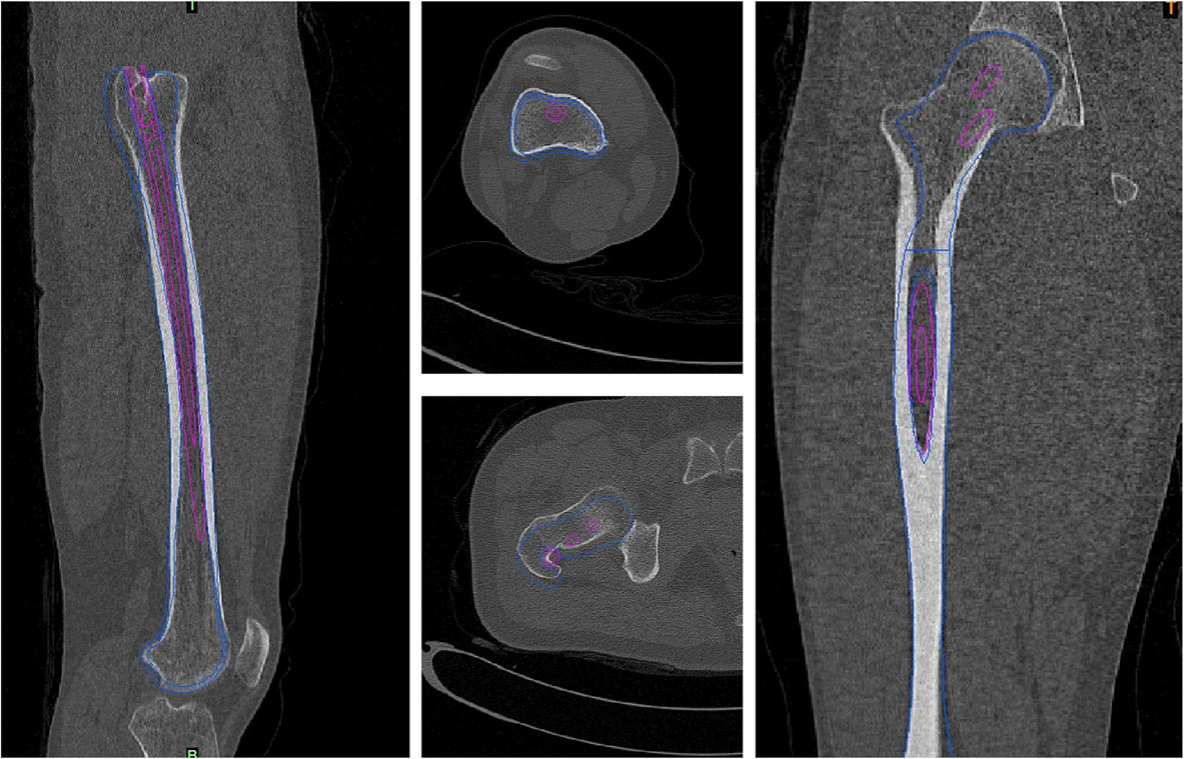
Striking between the head of nail and distal femoral cortex in Mimics14.0. Red: simulation of intramedullary nail; blue: simulation of femur

**Fig. 7:**
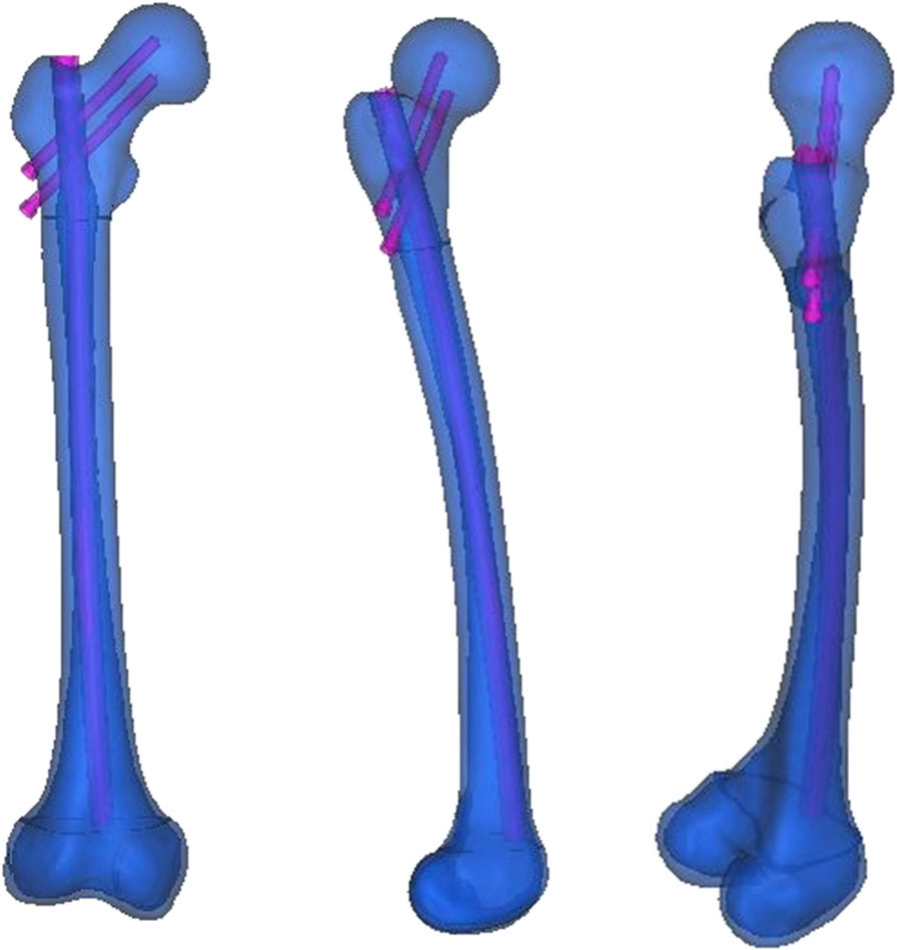
Striking between the head of nail and distal femoral cortex in Geomagic Studio 12.0

For the femur model whose radius of the femoral anterior bow is D, the tangent value of the femoral anterior bow (tan) will result in striking between the head of nail and distal femoral cortex (Figs. 6, 7).

The methods of calculating the radius and tangent value of femoral anterior bow are as follows:

Radius of the femoral anterior bow (D) = (AO^2^ + CO^2^)/2CO; and tangent value of femoral anterior bow (tan) = CO/AO,the corresponding central angle = 4∠CAO.

**Fig. 8:**
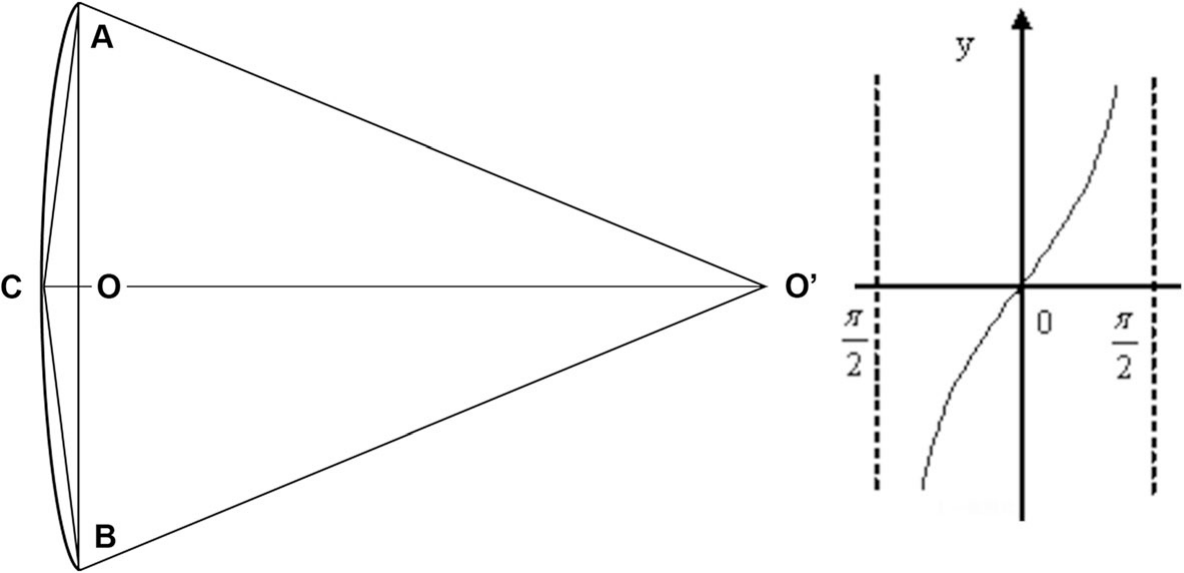
Tangent value of femoral anterior bow: CO/AO; D = (AO^2^ + CO^2^)/2CO

The tangent value increases progressively in the interval of 0° and 90° (Fig. 8). Upon comparing the radius and the tangent values, obtained from the preoperative X-ray of the contralateral femur, with the corresponding results, it was observed that CMN was not suitable in patients with shorter radius and bigger tangent value than the index.

## Results

Three types of right femoral cephalomedullary nails (ø10, 11, 12 mm × 350 mm; Smith-Nephew Co.) were involved in our study. The nail head collides with the distal femur anterior cortex when the diameter of the femoral intramedullary nail is 10 mm, radius of curvature (ROC) is 50.0243 cm, and tangent value of the femur anterior arch is greater than 0.140185, the corresponding central angle is 21.72°. The nail head collides with the distal femur anterior cortex when the diameter of the femoral intramedullary nail 11 mm and ROC is 52,5974 cm respectively, and the tangent value of the femur anterior bow is greater than0.133073, the corresponding central angle is 20.92°. The nail head collides with the distal femur anterior cortex when the diameter of the femoral intramedullary nail is 12 mm and ROC is 75.0552 cm respectively, and the tangent value of the femur anterior bow is greater than 0.092415, the corresponding central angle is 16.32°.

## Discussion

This study demonstrates a precise method in measurement associated with femoral anterior bow with finite element software, which avoid errors caused by individual difference and X-ray radiographic magnification base on calculating the ratio rather than length. The concept of femoral anterior bow tangent describes the extreme curvature when the CMN abut the anterior cortex at distal femur, and indicates mismatch between the intramedullary nail and femoral medullary cavity pre-operation.

Egol et al. [5] studied the anterior curvature of 948 femur specimens, and the value of radius of curvature (ROC) was found to be 120 ± 36 cm. A large mismatch was observed between the ROC of femurs and femoral nails with ROC value ranging from 186 to 300 cm (i.e., straighter than the adults femurs). Harper and Carson [6] determined the anterior radius of curvature of 14 femurs using a curve-fitting program with digital radiographs and reported that the radius ranged from 68 to 188 cm, with an average radius of 114 cm. The initial femoral middle section radius of curvature in this study is 130 cm.

The normal femur anterior bow acts as the force-line of the human body, but also hinders the femoral intramedullary nailing. Various parameters, including congenital, acquired nutrition, lifestyle, and work environment, exert significant effect on individual growth and development. Researchs demonstrated that there was no significant correlation between the radius of femoral anterior bow and age, however, individual differences in race and gender was found to influence the bow parameters; femur anterior bow radius in white population was smaller as compared to the black population, Asian population has the smallest femur anterior bow radius. Male femur anterior bow radius was significantly bigger than women; however, the left femur anterior bow radius is slightly larger than the right in 67 % of the female samples [5]. There is a linear relationship between the femur anterior bow radius and femur length. Results from the linear regression analysis showed that with every 1 cm increase in the length of femur, left femur anterior bow radius increases by 3.4 cm. The linear relationship was found to be non-affected with gender, age, race, BMI, and cortical bone thickness etc. [7] In many samples, no difference was observed between the bow radius of the medullary cavity of femur and the radius of the overall thigh cortex. [8] In this study, the femur models with different medullary cavities were established and the corresponding various femur bow curvatures were adjusted subtly using finite element software intending to cover most of individual characteristics comprehensively. The simulation of isthmus tunnel plug was achieved by accordant intramedullary reaming. Since the remained anterior cortex at distal femur was thin in the model, with the elimination of cancellous bone, the anterior cortical abutment with intramedullary nail head could get fewer individual differences. In other words, femoral models with uniform length but different medullary diameters, were bent to variational curvature. For the elaborate bending process, femoral model with same curvature corresponding to individual one were regard as scale pair.

Appropriate size of CMN is extremely important for femoral fractures [9]. For CMN, the end of CMN should reach the area between the plane located superior to the border of patella and 2 cm below it. For the feasibility and veracity, healthy femur measurement is the most wildly used process for CMN length decision peroperatively. Peroperative X-ray of the uninjured leg could be used for measurement, but it should be noted that the enlargement effect could neither be eliminated by measurement of the specimen nor by software-based survey of images. In this study, 350 mm CMN, ending in the plane located 1 cm below the superior border of patella, was selected. Simultaneously, the tail wasn’t allowed to extrude outside the top of great trochanter, and the two locking screws were driven to keep the femoral neck in place. In this study, the ratio rather than length was used to obtain the measurements in the lateral view. Accuracy was improved for neglection of the enlargement effect.

The femoral matrix and 3D projection of medullary cavity appeared like a long hourglass. The femoral canal isthmus and the stenosis were located proximally to the midpoint of the femoral non-cancellous bone area. [9, 10] Parameters like stenotic coronary diameter, local cortical thickness, intraoperative selection, and reamed operation play an important role in determining the intramedullary nail diameter. Based on the measurement results, it was inferred that the intramedullary nail diameter ranged between 10.7710 ± 1.1593 mm, intraoperative overreaming can increase the fixation stability, extent of reamed degree is generally in the range of 2–3 mm. In this study, we selected the 10 mm, 11 mm, 12 mm intramedullary nails corresponding to 11 mm, 12 mm, 13 mm medullary respectively. Intramedullary nails with different diameters were paired with corresponding reamed degree models in order to reduce the individual variations, meanwhile, the simulative reamed degrees were limit up to 3 mm.

This study established femurs with different bow by finite element software, and then simulated the insertion process and the final position of the nail. The tunnels were established by sections separately as follows, a) proximal part includes the 14 mm reamed tunnel from the entrance toward the medullary, b) the middle tunnel based on the initial medullary cavity combined with a reamed channel 1 mm thicker than the chosen nail diameter at the narrow area, c) the distal cancellous bone tunnel was processed as the medullary cavity. The whole passage is resembles the “long calabash” shape. The middle femur channel curvature was modulated in order to simulate different anterior bows of the femurs, but the proximal and distal.

Egol et al. [5] opined that anterior cortical penetration with intramedullary implants and insertion were mainly influenced by the location of the fracture, entry point, and curvatures of CMN. During fracture, involving the subtrochanteric, diaphyseal, or distal femur, insertional difficulty can be addressed by angulation at the fracture or overreaming. Distal roomy medullary provides sufficient space that not only decreases resistance, but also reduces the risk of anterior cortical penetration, which occurs when the large curvature anterior bow of the femur is intact as in case of intertrochanteric or subtrochanteric fracture. However, when the fracture occurs below the femoral isthmus, or in a superior-posterior position, the anterior cortex is intact. Forceful insertion leads to iatrogenic fractures and results in a large curvature anterior bow, which, in turn, makes the treatment difficult.

To sum up, the matching of the intramedullary nail radian with the human femur anterior bow radian could reduce the risk of insertional resistance and the cortex penetration at the distal femur. Preoperative evaluation of femur anterior bow could predict the challenges associated with nail insertion and postoperative pain and of high risk of fractures around the internal fixation. And then, plate devices or short nails were taken into account when the penetration was predicted.

Improving the intramedullary nail anterior bow radian is comparatively easier than the aforementioned influencing factors. Many scholars have demonstrated that the anterior bow radius of most commonly used medullary nail is bigger than femoral medullary cavity [4–6]. Furthermore, they proposed new designs to increase the curvature of intramedullary nails. However, it remains unclear as to whether it will go against with the three point contact principle of the initial design of the intramedullary nail. Can perfect radian provide the best mechanical support? These queries require further biomechanical experiments and studies. The purpose of this experiment was to propose a convenient measurement and calculation method to predict the intraoperative and postoperative complications. Not all patients should be suitable for using the new intramedullary nail, but whom we forecast mismatch between their femoral curvature and current intramedullary nails.

Since the tangent value of the femur anterior bow curvature was accurately related to the femur anterior bow, the effect of X-ray radiographic magnification was eliminated by calculating the ratio of the measured value, radian, and angle size. We assumed that femurs with same bow curvature but different length as equal proportional models, at the same time, simulate medullary cavities with diverse diameters, so we endeavored to sweep up the most individuals, but there are still errors.

## Conclusion

A novel assessment of femur anterior bow curvature, the concept of femoral anterior bow tangent, provided a more accurate calculation method. It could be used not only for better preoperative planning and treatment, but also for inclusion criteria of the new nail designs.

Future studies may include determining the relevance of clinical practice and quantity of data calculation and assessing the practicality of the concept of femoral anterior bow tangent on the preoperative evaluation for cephalomedullary nailing of the femur.

## Abbreviations

AP: Anteroposterior
CMN: Cephalomedullary nail
CT: Computed tomography
ROC: Radius of curvature
Tan: Tangent value of femoral anterior bow
